# Microstructure and Thermal Analysis of Metastable Intermetallic Phases in High-Entropy Alloy CoCrFeMo_0.85_Ni

**DOI:** 10.3390/ma14051073

**Published:** 2021-02-25

**Authors:** Zihui Dong, Dmitry Sergeev, Michael F. Dodge, Francesco Fanicchia, Michael Müller, Shiladitya Paul, Hongbiao Dong

**Affiliations:** 1 School of Engineering, University of Leicester, Leicester LE1 7RH, UK; zihui.dong@le.ac.uk (Z.D.); Michael.Dodge@twi.co.uk (M.F.D.); shiladitya.paul@le.ac.uk (S.P.); 2 Forschungszentrum Jülich GmbH, Institute of Energy and Climate Research (IEK-2), Leo-Brandt-Strasse 1, 52425 Jülich, Germany; d.sergeev@fz-juelich.de (D.S.); mic.mueller@fz-juelich.de (M.M.); 3 TWI Ltd, Granta Park, Great Abington, Cambridge CB21 6AL, UK; francesco.fanicchia@twi.co.uk

**Keywords:** high-entropy alloy, thermodynamics, microstructure, phase evolution

## Abstract

CoCrFeMoNi high entropy alloys (HEAs) exhibit several promising characteristics for potential applications of high temperature coating. In this study, metastable intermetallic phases and their thermal stability of high-entropy alloy CoCrFeMo_0.85_Ni were investigated via thermal and microstructural analyses. Solidus and liquidus temperatures of CoCrFeMo_0.85_Ni were determined by differential thermal analysis as 1323 °C and 1331 °C, respectively. Phase transitions also occur at 800 °C and 1212 °C during heating. Microstructure of alloy exhibits a single-phase face-centred cubic (FCC) matrix embedded with the mixture of (Co, Cr, Fe)-rich tetragonal phase and Mo-rich rhombohedron-like phase. The morphologies of two intermetallics show matrix-based tetragonal phases bordered by Mo-rich rhombohedral precipitates around their perimeter. The experimental results presented in our paper provide key information on the microstructure and thermal stability of our alloy, which will assist in the development of similar thermal spray HEA coatings.

## 1. Introduction

Thermal spraying is a generic term for a group of processes in which metallic, ceramic, cermet, and some polymeric materials in the form of powder, wire, or rod are fed through to a torch or gun with which they are heated to near or somewhat above their melting point [1]. The resulting molten or nearly molten droplets of material are accelerated in a gas stream and propelled to form a coating on the substrate. On impact, the droplets flow into thin lamellae, overlapping and interlocking as they solidify. More recently, thermal spraying has been recognized as a key process for the synthesis of specialized coatings and materials. It offers the ability to create free-standing structures for net-shaped manufacture of high-performance ceramics, composites and functional graded materials. It is also used for the rapid-solidification synthesis of specialized materials.

However, due to the high temperature involved in thermal spray, the molten material is likely to be associated with vaporization or evaporation of elements [2,3,4] leading to phase instability in multicomponent alloys as a consequence of abrupt change in chemical composition. Therefore, the materials used for thermal spraying require good thermal stability and durability to achieve good performance of final sprayed surfaces. Under such considerations, high-entropy alloys (HEA) are potentially promising candidates for thermal spraying due to good thermal stability, high hardness and mechanical strength, and excellent corrosion/oxidation resistance [5,6,7,8]. Hence, assessments of thermal stability of HEA system will enable practical applications of materials regarding improved understanding for phase evolution and compositional modulation, which will envisage implementation in thermal spraying.

Mo-based intermetallics have a good combination of physical and mechanical properties, including thermal stability. Consequently, Mo is often considered as a precipitation hardening element in modern alloying design, due to its capability to form hard intermetallics with Co, Cr, Fe, and Ni [9]. Therefore, CoCrFeMoNi alloy is allocated as a novel combination with respect to HEA designs for thermal spraying applications. According to previous research, the CoCrFeMoNi alloys exhibit good hardness and phase stability at temperatures above 700 °C [10] as well as good corrosion resistance in form of gas atomized powder [11]. X-ray diffraction pattern of as-cast CoCrFeMo_0.85_Ni consists of a stable FCC structure, BCC and the small amount of µ phase while the annealed CoCrFeMoNi HEA at 800 °C shows FCC peak and µ phases. The FCC matrix tends to exhibit good thermal stability. However, there are insufficient data on the thermodynamic properties which are required to further assess the phase stability and high-temperature performance near its solvus temperature. In addition, experimental results studied by Otto et al. [12] suggested that enthalpy and non-configurational entropy have great influence on phase stability in equiatomic, multi-component alloys. Hence, in order to investigate the thermodynamic properties of CoCrFeMoNi HEA, detailed thermal analysis is required to obtain valid information for assessing performance and phase stability at the upper end of the temperature range of interest.

In this paper, thermal analysis of the CoCrFeMo_0.85_Ni HEA powder was carried out via differential thermal analysis (DTA) and differential scanning calorimetry (DSC) to assess the thermodynamic properties of HEA, including the solidus, liquidus and phase transition temperatures. With detailed characterization of microstructures in the DTA sample, the critical temperatures determined in thermal analysis can be correlated with microstructural features to examine phase formation and formation of precursor states from compositional modulations in HEA. This will provide fundamental understanding on intermetallic strengthening mechanism and phase stability for processing of HEAs.

## 2. Materials and Methods

The samples of CoCrFeMo_0.85_Ni were gas atomized from high purity Co, Cr, Fe, Mo, and Ni in the form of as-supplied powder, which has approximate size of 24 µm. The nominal composition of the CoCrFeMo_0.85_Ni HEA are provided in Table 1, showing equimolar fraction among CoCrFeNi in this HEA. The consistence between nominal and as-received composition of powder also has been confirmed via following semi-quantitative measurements.

The thermal analysis was then performed using two Netzsch devices (Netzsch, Selb, Germany) (DSC 404 and STA 449 F3), which have two different types of sample holders and crucibles. The measurements of samples were repeated as three heating and cooling cycles adopting 5 K/min interval up to maximum temperature of 1400 °C. One of the CoCrFeMo_0.85_Ni samples was measured by DSC404 (initial weight was around 129.3 mg) in Al_2_O_3_ crucible and under Ar-atmosphere (at the beginning the heating zone of the device was evacuated). The same temperature profile was applied for baseline measurement with empty crucible, which was used to exclude the artificial signals of the sample holder. Two measured cycles are referring to DTA and DSC with baseline in Table 2, which lists the mass change of each sample with an accuracy of 0.1 mg demonstrating negligible mass changes for both thermal measurements (0.02% increase in DTA cycle and a 0.61% increase, up to 130.1 mg recorded in the DSC measurement). The interrupted DTA experiment at 900 °C was additionally performed for characterization of initial phase transition and corresponding microstructures at the first heating stages.

In order to further inspect the phase stability of heated and interrupted HEA samples in DTA with respect to the original powder, samples were mounted in conductive resin and polished, using standard metallographic preparation techniques. The microstructures were then characterized using a Zeiss Sigma (Zeiss, Oberkochen, Germany) field emission gun scanning electron microscope (FEGSEM) equipped with energy-dispersive X-ray spectroscopy (EDX) detector. Semi-quantitative EDX point and line-scan analysis was conducted across salient microstructural features. Phase and crystallographic information were determined by electron backscatter diffraction (EBSD).

## 3. Results

**Figure 1:**
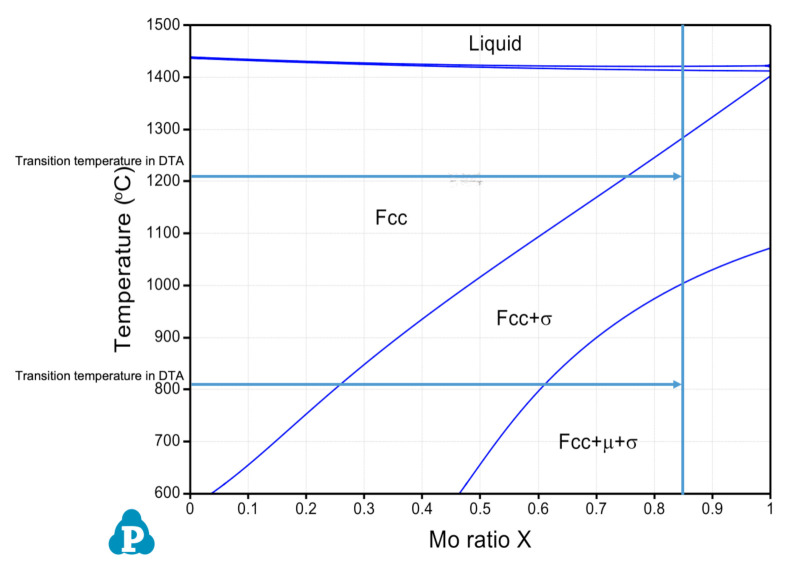
Pseudo phase diagram of (Co, Cr, Fe, Ni)–Mo versus mole fraction of Mo (i.e., Mo_0.85_) showing co-existence of predicted phases in terms of increased Mo content.

CALculation of PHAse Diagrams (CALPHAD) approach [13] is typically employed in alloy design to predict microstructures as a product of elemental composition and temperature. In order to predict which phase constituents form in the CoCrFeMoNi system, the PanHEA database [14] contained within Pandat^®^ was used. A pseudo phase diagram for the (CoCrFeNi)-Mo system versus an ascending temperature scale, calculated under thermal equilibrium conditions, is provided in Figure 1. For the designated compositions of CoCrFeMo_0.85_Ni HEA, i.e., ratio of (CoCrFeNi)-Mo as 1:0.85, the corresponding phases are predicted to contain a single-phase FCC structure at temperatures above 1280 °C, a mixture of FCC and σ phase in range of 1000 to 1280 °C, and a mixture of FCC, σ and µ phase below 1000 °C. Hence, following calorimetry experiments (two results are attached in advance as a result comparison) will quantitively confirm the transition temperatures of multiple phases predicted in the CoCrFeMo_0.85_Ni HEA under CALPHAD calculations.

**Figure 2:**
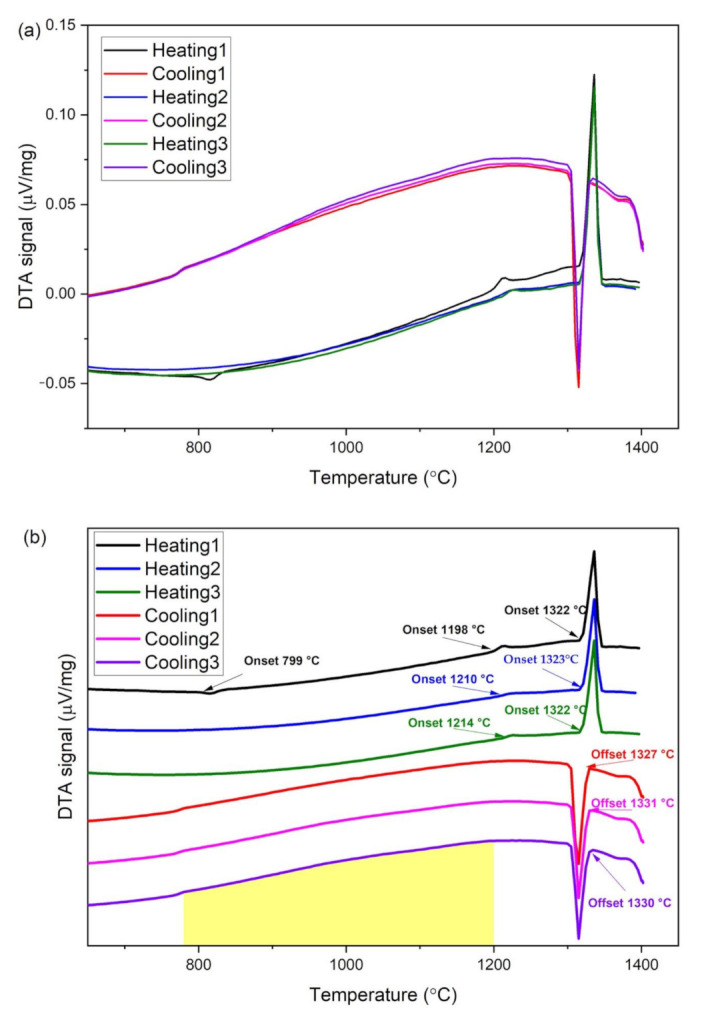
(**a**) DTA curve within same scale and (**b**) enlarged DTA curves highlighted phase transition temperatures of measured CoCrFeMo_0.85_Ni HEA from consecutive heating-cooling cycles.

The raw DTA curve measured from CoCrFeMo_0.85_Ni powder is shown in Figure 2a, also enlarged as individual curves for optimized visualization in Figure 2b. Since the DTA sample was heated from a gas atomized powder, which was prepared by rapid solidification, there are substantial difference in thermal resistances for original powder and melted bulk alloy, due to far slower solidification rate during cooling cycles of DTA experiments. Hence, there is always a slight discrepancy between the first cycle of a sample and repeating cycles due to heat absorption difference in sample status, i.e., the powder and solidified sample in this DTA process. The phase transition temperatures were obtained from onset of a kink in the DTA curve, while the solidus and liquidus temperatures can be derived from onset of the heating curve and offset of the cooling curve (all transition temperatures are indicated by arrows in Figure 2b). Further details of the analytical method can be found in this article [15]. Hence, it is evident that the transition temperatures of the CoCrFeMo_0.85_Ni HEA are at *T* = 800 °C and 1212 °C, where sharp kinks are observed in the first DTA curve (black line in Figure 2). The formation of intermetallics occurs during exothermal process at 800 °C, tends to be the interaction between µ and σ phase, whereas dissolution ascribes to endothermic reaction at 1212 °C. As temperature continues to increase, the solidus and liquidus temperatures (mean temperature of cycle 2 and 3) are found to be 1323 °C and 1331 °C, respectively. Hence, the primary solidification path in terms of freezing the FCC phase is determined to be 1200 °C < T < 1320 °C. Furthermore, the steeper gradient of cooling curve shows the precipitation window in the temperature range of 780 °C and 1200 °C (shaded area of cooling curves in Figure 2b). The obtained data confirmed initiative temperatures for melting and solidification of the CoCrFeMo_0.85_Ni HEA. All critical temperatures from DTA and DSC measurements are summarized in Table 3.

By considering the Cr–Mo–Ni [16] and Cr–Fe–Mo [17] ternary phase diagrams, the eutectic reaction occurs at 1275 °C and 1345 °C respectively, indicating a selective temperature range for phase transition which is consistent with the heating exchange curve determined using the DTA/DSC technique. It is also worth mentioning that the phase diagram predicted in Figure 1 shows a single FCC phase for CoCrFeMo_0.85_Ni alloy at temperature above 1280 °C, which has a disagreement with temperatures obtained in DTA measurements. The CALPHAD method may need further assessment for transition temperature of intermetallics formation for this HEA.

**Figure 3:**
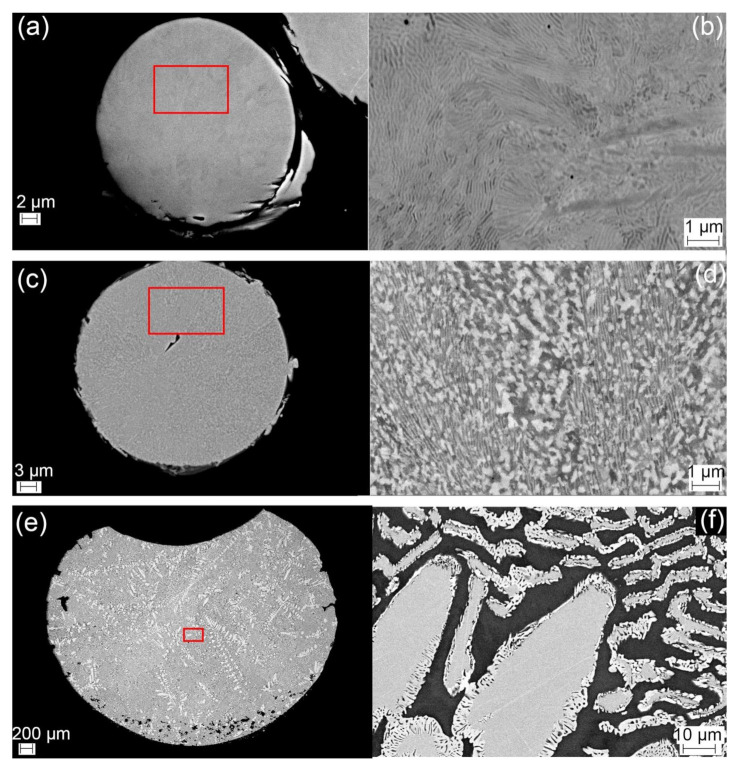
Backscattered electron images of (**a**) as-received powder, (**b**) a highlighted region of the as-received sample in high magnification, (**c**) powder from the interrupted DTA sample at 900 °C, (**d**) a highlighted region in interrupted sample in high magnification, (**e**) the post-DTA sample in low magnification, (**f**) a highlighted area in post-DTA sample in high magnification.

The detailed microstructures of HEA samples are illustrated in Figure 3 presenting microstructures in as-received powder, initial phase transformation and final solidified sample. Table 4 enumerates the chemical compositions of CoCrFeMo_0.85_Ni HEA measured from sites of interest using EDX. First of all, there is a good agreement between nominal and chemical composition of the alloy in as-received state confirming the consistency of semi-quantitative EDX measurements. In the as-received powder, there is minor elemental segregation across the powder particle in Figure 3a, whilst the detailed SEM image in Figure 3b shows fine alternating layers which have some similarity to the morphology formed via spinodal reaction in Al–(Co)–Cr–Fe–Ni HEA [18]. Further analysis on the chemical composition of the alternating layers is needed to clarify the reaction for the formation of the phases. Figure 3c shows low magnification backscattered image of interrupted DTA samples at 900 °C. Grain structure in the DTA sample is coarsened comparing to that in as-received samples and growing direction toward the center of a powder can be observed macroscopically in Figure 3c. Further high magnification image in Figure 3d shows a mixture of lamellar and particle-like microstructures in the HEA powder, which evidently proves the initiation of phase formation at around 800 °C corresponding to the kink in the first cycle of DTA curve illustrated in Figure 2b. After completion of the full thermal cycles in the DTA experiment, a typical dendritic microstructure was observed. The low magnification image of Figure 3e, representing the solidified HEA sample of ~4 mm size, shows arbitrary growth of dendrites with secondary or ternary morphology. These features are microscopically examined at higher magnification in Figure 3d observing coarsened dendrites. The most remarkable feature of the DTA sample is the mixture of two complex phases which consist of (Co, Cr, Fe)-rich tetragonal phase (presumably σ phase) in dendrite core surrounded by a fringe of Mo-rich rhombohedral phase (presumably μ phase) along the perimeter, as denoted by an obvious contrast difference in the backscattered electron image. The additional EDX results (see Table 4) confirm that the FCC matrix is depleted in Mo, while the dendritic core is enriched in Mo and depleted in Ni, particularly in dendrite tip where is more enriched in Mo. The distribution of elements exhibits similar results to what reported in numerous studies of superalloys and has been attributed due to sluggish diffusion of Mo during solidification, which tends to partition to the interdendritic regions. Chemical compositions of different zones reveal approximately equal distribution of Co, Cr, and Fe in FCC matrix indicating good phase stability of CoCrFe-based matrix after thermal cycles, with very minor segregation of constituent elements.

**Figure 4:**
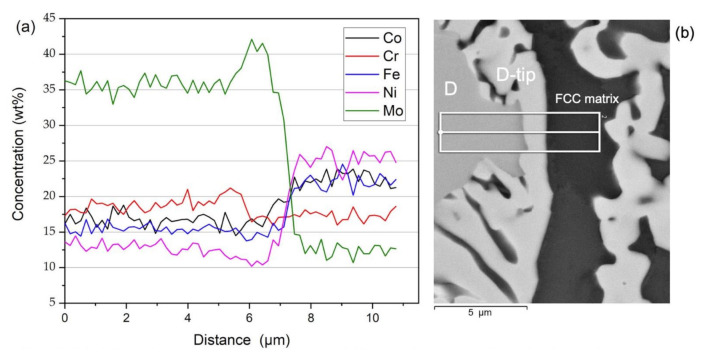
(**a**) Variation of elemental concentration in weight percent, (**b**) backscatterred electron image on scanned site for linescans area across dendrite and interdendrite showing enrichment and depletion of elements among different phases.

In order to further examine and identify the observed phases in detail for DTA sample, EDX linescans were performed across a distance of approximately 11 µm to reveal any chemical gradients across the area of interest, as shown in Figure 4a. The microstructures in Figure 4b showed distinct phases at the interface of the dendritic areas, where the periphery of dendrite has the brightest contrast. The concentration of Mo remains at a level in the dendrite and peaks in the fringe of dendrite (labelled as D-tip) while FCC matrix is deficiency in Mo (less than one third of that inside the dendrite). In contrast, the concentration of Ni is depleted in dendrite whereas it is one thirds higher in the FCC matrix. In general, data from linescan reveals major features of elemental distribution confirming deficiency of Mo and equal distribution of Co, Fe, and Ni in FCC matrix, alongside Mo enrichment and Fe and Ni depletion in the dendrite core. This indicates a relatively abrupt change in compositions among the observed phases at interface of the dendritic region.

**Figure 5:**
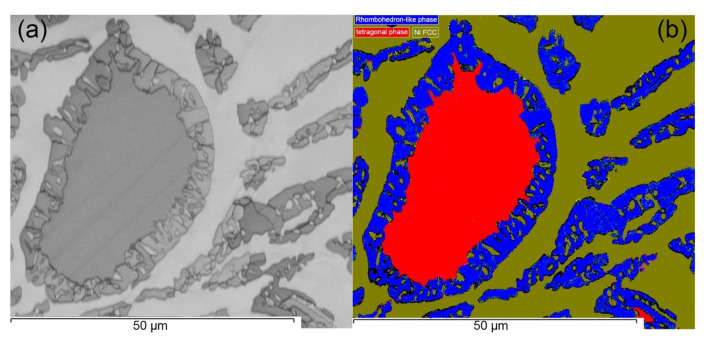
(**a**) Band contrast image of the post-DTA sample (Figure 3e), (**b**) electron backscatter diffraction (EBSD) phase distribution map of the full cycle DTA sample showing FCC phase embedded with tetragonal phase and rhombohedron phase.

Further phase characterization was performed using EBSD in the region around the bulk dendrite and FCC matrix to differentiate observed phases based on crystallography as shown in Figure 5. It is clear from the phase map in Figure 5b that the background matrix exhibits a single-phase FCC structure dominated by Ni. The bulk of embedded dendrite matches the tetragonal structure while corresponding peripheral area as well as dispersed particle in adjacent regions is consistent with the pattern of rhombohedral structure. Based on measured compositions of the HEA in this study and corresponding literature studies on the CoCrFeNiMo_0.85_ HEA [10] and the ternary Fe–Cr–Mo system [19], the crystal structure of dendrites corresponds to tetragonal σ structure surrounded by rhombohedral µ phase. 

## 4. Discussion

### 4.1. Phase Evolution in CoCrFeMo_0.85_Ni High-Entropy Alloy

In a similar way to characterize solidification path like Ni-base superalloys [20,21], the formation of A1-type FCC solid solution matrix in HEA is derived via thermal analysis attributing to a high mixing entropy effect, which reduces the Gibbs free energy of mixing, thus facilitating the formation of solid solution upon solidification. Each element in CoCrFeMoNi HEA has different partitioning towards dendrite and interdendrite that are associated with local imbalance of concentrations. Especially for Mo, element with the highest melting point among the five constituents in this HEA, rejection of solute to liquid leads to Mo partitioning towards interdendrites, where is the last portion to solidify during cooling. Eventually, the solidified CoCrFeMo_0.85_Ni alloy after DTA cycles forms microstructure consisting of a more homogenized FCC matrix embedded with Mo-rich intermetallics, where central tetragonal phase is surrounded by rhombohedral phase in periphery. In addition, the coarsening of Mo-rich interdendrites was observed at different stages of the DTA experiment indicating that the temporal growth of Mo-rich metastable phase initiated from 800 °C. The formed particles possess local chemical enrichment of elements such as Mo and Cr. Once the level of enrichment is favorable as precursor, chemical fluctuation presented in the miscible blend will subsequently occur initiating an unstable process alongside thermal effect and lead to demixed morphologies, since segregation is ubiquitous at interfaces of dendric and interdendritic regions [22].

Based on the pseudo binary phase diagram of (CoCrFeNi)-Mo obtained by CALPHAD method in Figure 1, the CoCrFeMo_0.85_Ni HEA consists of two multi-phase regions from FCC+σ+µ towards FCC+σ region with respect to an increasing temperature. Thus, the formation and dissolution of metastable phases during ramp up is plausible according to detected peaks in DTA curves. In this context, the appearance and disappearance of peaks are regarded as initiation temperature for different metastable phases, which has been studies in terms of several phenomena including spinodal decomposition, supersaturated solid solution and precipitation [23,24,25,26]. Metastable state in alloy has been comprehensively reviewed by Cahn and Greer describing any state with a Gibbs free energy higher than the lowest value corresponding to stable equilibrium, microstructural manifestation characterized by metastable phase appear metastably under different conditions of composition, temperature or pressure [27]. Metastable phenomena, such as spinodal decomposition and precipitation of intermetallics, receive a great upsurge in study of various HEA systems. Several research papers [9,10,28] showed good combination of strength and ductility in CoCrFeNiMo HEA after annealing owing to formation of σ and µ phases. There are substantial increases of hardness and strengthening after ageing ascribe to intermetallics. The increase of hardness is due to lattice distortion induced by larger Mo atoms. Cai et al. [29] also found that Mo-rich intermetallic inhibits motion of dislocation and increase strength in annealed CoCrFeNiMo HEA. The compositional modulation was observed for Mo and Ni in the interfacial plane of HEA showing certain similarity with previous publications in terms of Fe–Cr–Mo and the bulk of Fe–Cr–Co and Fe–Cr alloys [19,30,31,32]. Furthermore, μ particles was found mainly grow at boundaries indicating additional strengthening effect attributing to intermetallic [33]. Meanwhile, our results showed that the mixture of metastable phase initiates at around 800°C in powder CoCrFeMoNi HEA associated with exothermal effect, which appears to be a short-term phenomenon once sufficient thermal energy is applied. Based on aforementioned calculated phase diagram and other studies, transformation between σ and µ phase is attributing to exothermal process around 800 °C threshold as a result of an increased temperature [10,28]. It is also noticed that the melted HEA exhibits different heat absorption coefficient in repetitive DTA cycles comparing to original powder form (i.e., signal difference in DTA cooling curves), thus, this denotes interaction of metastable phases at 800 °C due to exothermal effect in powder form can be eliminated by high temperature treatment in reaching a more homogenized status. Additionally, an analogous phenomenon was reported for thermal exposure less than 100 h, which is insufficient duration to allow secondary or ternary phase to form in HEA [34], the phase stability is believed to depend on exposure times for reaching equilibrium state of a HEA constituent. Hence, the kinetics effect tends to play a determining role in HEA system due to sluggish diffusion kinetics [35]. Since the stabilization of σ phase in CoCrFeMo_0.85_Ni HEA remains a gradual process in reaching its equilibrium state, which tends to be too slow to be observed in DTA experiments. As being deduced, transition of metastable phases is more or less depending on kinetic effect. Segregation driven by diffusivity will be a major process as a long-term effect before initiation or completion of phase separation. Therefore, Mo with relatively slower diffusion rate in FCC matrix eventually segregates to form µ phase known as a precursor state. The thermal stability of CoCrFeMo_0.85_Ni HEA depends on the extent of metastable phases in transition period in control level of Mo segregation. 

Since absorption and spinodal decomposition are known precursor states to phase transition, spinodal fluctuation driven by segregation act as precursor to form a new phase [36]. Phase separation through spinodal decomposition has been found as common phenomena in the bulk of HEAs [37], while uphill diffusion and compositional modulation is recently reported in terms of similarity with respect to spinodal decomposition [38]. Although HEA consists of multiple equiatomic or near equiatomic elements exhibiting excellent distribution of elements in as-received raw samples, there still is existence of subtle compositional imbalance in local regions where tend to be initiation sites for phase transition (as growth or nucleation of lamellar-like microstructure at early stage in ramp up to spinodal microstructure). Considering concentration gradient in a solution, unification/homogenization has the effects on dilution of solution with higher elemental concentration. Spinodal decomposition is a process by which a thermodynamic unstable, virtual homogeneous solution can transform within a miscibility gap to a mixture of phases that are close to equilibrium compositions.

It is interesting to mention an assumption by He et al. [25] that the formation of Cr-rich σ phase is induced by Al addition attributing to lattice distortion in CoCrFeNiAl_0.1_ alloy. Being the largest one among four elements, size misfit of Mo-rich particles produces coherency strains [39]. Moreover, larger lattice strain in powder form than the one in as-cast form leads to higher theoretical strength enhancement [40]. It was also discussed by Liu et al. that the atoms Mo segregation and particle precipitation strengthening in the as-cast Mo_0.2_ and Mo_0.3_ are more effective than solid solute strengthening [28]. Hence, pronounced solid solution strengthening can be achieved by lattice distortion (due to size misfit of atoms) impeding dislocation movement. Mo segregation and particle precipitation also relaxed the lattice dilation. Ultimately, Mo-rich intermetallic tends to act as strengthening phase for property improvement when the incorporating Mo element remains at a relatively low level to CoCrFeNi matrix, i.e., solute element. Apart from aforementioned, the addition of Mo to CoCrFeNi HEA alloy exhibits higher metastable stability temperature than CoCrFeNi alloy (thermally metastable at 750 °C) showing the effect of Mo in improving metastable status [33].

### 4.2. The Coexistence of σ and µ Phases

In accordance with obtained results above, the microstructure of CoCrFeMo_0.85_Ni alloy is confirmed with mixture of three phases showing consistent features reported in previous studies of annealed CoCrFeNiMo_x_ HEA [9,28] and CorCrFeMoNi for coating applications [41,42,43]. Two phases are formed during short-term exposure of HEA to high temperature, which is consistent with previous studies showing occurrence of phase after 1 h although the HEAs were annealed at a lower temperature. Hence, it can be deduced that the formation of phase is attributing to short-term exposure as confirmed by interrupted DTA experiment. The formed phases in CoCrFeNiMo HEA are likely to be tetragonal σ phase and rhombohedral µ phase as predicted in Figure 1.

Considering the process of solidification, it reacts as the transport of solute between the solid and the liquid phases. In HEA, the elements that partition to solid are Co, Fe and Ni, while Mo is likely to partition to liquid. Simultaneous existence of two phases can be deduced by cooperative diffusion of constituent atoms depending on the order of elemental partitioning towards the interface of phases. Upon solidification, different rate of diffusivities among five elements lead to the order solidification due to concentration in the solution, dendrite and large σ particle is initially solidified whereas Mo containing solidified at the last stage eventually trapping portion of formed phases in between (Figure 2f). The order of phase transition is defined by the lowest-order derivative of the Gibbs energy that changes discontinuously at the transition [44], second derivative of the free energy determines the occurrence of spinodal decomposition [45]. Since spinodal decomposition is a spontaneous process, no free energy barrier must be overcome. If spinodal decomposition occurs, a system is able to bypass nucleation long enough to penetrate into the unstable region of the phase diagram [46]. The SEM image showed a progressive penetration of Mo-rich µ phase as a consequence of coarsening. The growth of intermetallic precipitates in HEA is often associated with thermal effect, while a period of annealing applied results in larger size of precipitates, such as Mo-rich µ phase [33] and Cr-rich σ phase [24]. Furthermore, there are extensive studies of Mo containing alloys [47,48,49] associated with spinodal decomposition. Hence, destabilization of the σ particle leads to local compositional imbalance, which is eventually attributing to microstructural evolution associated with composition modulation. The Gibbsian enrichment in solutes shifts the local thermodynamic state of the interface into a spinodal regime in the bulk of Cantor alloys with further uphill diffusion inside the grain boundary plane during ageing [38]. Moreover, Cr and Mo addition affects interfacial Gibbs free energy, i.e., smaller Gibbs free energy with increase of Cr concentration and vice versa. Hence, it forms the precursor state in developing new phases with thermal energy applied. The mixture of µ and σ phase in interdendrites is indicating simultaneous existence subjecting to precipitation hardening for CoCrFeMo_0.85_Ni HEA, which can be strengthened by those intermetallic particles in the annealed CoCrFeMoNi alloy [29] as long as the phases remain at a desirable and controllable amount in HEA.

**Figure 6:**
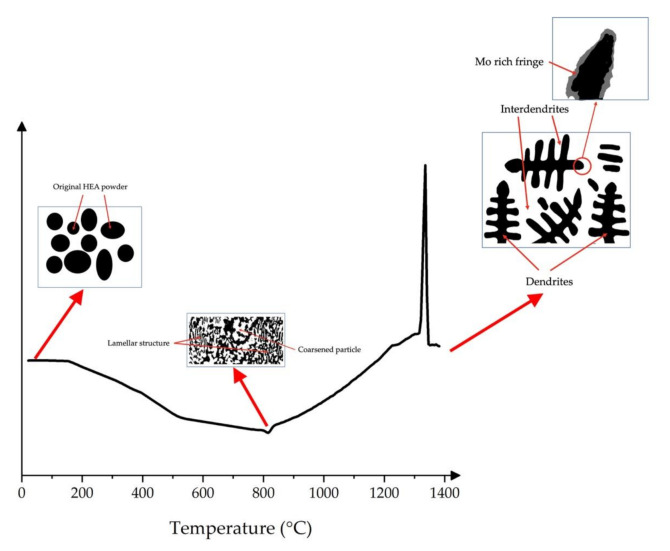
Schematic of the phase evolution within this HEA consisting of the initial powder status, transition state and final microstructure solidified from melting.

Overall, the schematic of the phase evolution in CoCrFeMo_0.85_Ni HEA is deduced as illustrated in Figure 6 visualizing the progress of intermetallic formation. At the initial stage of ramp up, elements in the HEA powder commence to preferentially partition in metastable phases, which is a precursor state of spinodal phase showing at intermediate temperatures. As metastable transition is a long-term process in CoCrFeMo_0.85_Ni HEA, the evolution of metastable phase is driven by segregation attributing to spinodal decomposition despite a long-term thermal effect attributed. During the cooling, Mo is partitioning toward interdendrites where solidified at the last stage leading to Mo-rich microstructure at dendrite tip. Occurrence of spinodal decomposition in unstable solute results in phase migration by penetrating into the unstable region, which initiates the growth and coarsening of Mo-rich µ phase into σ phase. With further thermal effect applied, metastable σ phase is likely to be continuously consumed by coarsened µ phase or visa verse depending on extent of Mo and Cr enrichment until local thermodynamic equilibrium is reached.

Further detailed thermal analysis should be proceeded to derive the remaining thermodynamic properties, such as partial pressure, activity etc., in order to comprehensively inspect and quantify fundaments of this novel CoCrFeMo_0.85_Ni HEA in terms of practical applications as a high-temperature material. Apart from that, the formation of metastability has initiated novel and promising concept in terms of thermodynamic weakness in phases for leading to higher strength and damage tolerance. The most recent metastability alloy design (MAD) concept proposed by Raabe et al. [50] aims to tune compositional, thermal, and microstructure of metastable phase states for triggering diffusive or athermal transformation mechanisms. Moreover, the exceptional mechanical behavior is associated with the nature of particle distributions [28]. Formed particles, which are precipitated out from the supersaturated FCC matrix by thermal effect, are essentially discrete as reported becoming finer due to slow diffusion process in HEA [35]. Considering above aspects, further investigation of intermetallic phase will facilitate conclusive understanding associated with exploitation of atomic arrangement and orientation to reveal the interfacial structure for optimal HEA design.

## 5. Conclusions

In conclusion, thermal analysis of CoCrFeMo_0.85_Ni HEA via DTA, DSC method and corresponding characterization of its microstructure are presented. Solidus and liquidus temperatures were found as 1323 °C and 1331 °C, respectively. Two transition temperatures were also observed at 800 °C and 1212 °C confirming the formation of new phases at corresponding temperatures. A typical dendritic structure has been observed from CoCrFeMo_0.85_Ni HEA after consecutive cycles of the DTA experiment. The FCC matrix is embedded with complex multiple phases which are the mixture of (Co, Cr, Fe)-rich tetragonal phase and Mo-rich rhombohedral phase. The detailed morphologies of two intermetallics show tetragonal phases (deduced as σ phase) in dendrite core is surrounded by Mo-rich rhombohedron precipitates (deduced as µ phase) along their perimeter. The mixture of µ and σ phase in interdendrites indicates that precipitation occurs in CoCrFeMo_0.85_Ni HEA, which can play a role for optimize properties of HEA through precipitation hardening.

## Figures and Tables

**Table 1:** Nominal compositions of as-received CoCrFeMo_0.85_Ni high entropy alloy (HEA).

CoCrFeMo_0.85_Ni HEA	Al	Co	Cr	Fe	Mo	Ni
wt%	Max 0.10	19.2 ± 0.2	16.94 ± 0.2	18.19 ± 0.2	26.55 ± 0.2	19.12 ± 0.2
at%	Max 0.10	20.62 ± 0.2	20.62 ± 0.2	20.62 ± 0.2	17.52 ± 0.2	20.62 ± 0.2

**Table 2:** Weight changes of differential scanning calorimetry (DSC) and differential thermal analysis (DTA) samples showing negligible mass losses.

Measurements Cycles	DTA	DSC with Baseline
Mass before (mg)	428.75	129.3
Mass after (mg)	428.76	130.1

**Table 3:** Detected temperatures (°C) of thermal effects of the studied samples CoCrFeMo_0.85_Ni HEA.

Measurements Cycles	DTA	DSC with Baseline	Interrupted 900 °C DTA
1st heating (°C)	799 (exo *) 1198 1322	803 (exo *) 1197 1319	801 (exo *)
1st cooling (°C)	1327 1321	1320	-
2nd heating (°C)	1210 1323	1221 1318	-
2nd cooling (°C)	1331 1323	1319	-
3rd heating (°C)	1214 1322	1233 1318	-
3rd cooling (°C)	1330 1323	1319 1169	-

* exo denotes exothermal effect observed on the heating curves. All other temperatures corresponding to endothermal exothermal effects for heating and cooling curves, respectively.

**Table 4:** Nominal and measured chemical compositions of CoCrFeMo_0.85_Ni high-entropy alloy: Raw powder and DTA samples (wt%).

	Co	Cr	Fe	Mo	Ni
Nominal	19.2 ± 0.2	16.9 ± 0.2	18.2 ± 0.2	26.6 ± 0.2	19.1 ± 0.2
As-received EDX (raw powder)	19.28 ± 0.24	17.10 ± 0.18	17.93 ± 0.2	27.34 ± 0.3	18.35 ± 0.24
DTA FCC matrix	22.23 ± 0.24	16.03 ± 0.17	21.09 ± 0.21	16.32 ± 0.26	24.33 ± 0.26
DTA central dendrite (σ)	16.42 ± 0.20	18.62 ± 0.19	15.25 ± 0.20	36.72 ± 0.31	12.99 ± 0.22
DTA dendrite tip (µ)	16.13 ± 0.23	17.51 ± 0.18	13.86 ± 0.19	42.49 ± 0.31	10.01 ± 0.21

## Data Availability

Data sharing not applicable.
